# Barbed Sutures and Their Potential Role in Reducing Inflammatory Reaction After Cesarean Delivery: A Single-Center Experience

**DOI:** 10.7759/cureus.44094

**Published:** 2023-08-25

**Authors:** Kohei Kitada, Yasushi Kurihara, Mie Tahara, Akihiro Hamuro, Takuya Misugi, Akemi Nakano, Masayasu Koyama, Daisuke Tachibana

**Affiliations:** 1 Obstetrics and Gynecology, Osaka Metropolitan University Graduate School of Medicine, Osaka, JPN

**Keywords:** polydioxanone suture, surgical site infection, uterine closure, barbed suture, cesarean delivery

## Abstract

Objectives

The aim of this study was to investigate the short-term outcomes of knotless barbed sutures used for both closures of myometrium and subcuticular tissues in patients with various operative indications and who underwent cesarean delivery (CD) in a single tertiary center.

Materials and methods

A retrospective cohort study was conducted, and the patients were divided into two groups. The barbed suture group consisted of patients who underwent CD using barbed sutures for uterine closure (0 Stratafix® Spiral PDS Plus, Ethicon, Somerville, NJ, USA) and subcuticular closure (4-0 Stratafix® Spiral PDS Plus). The non-barbed group consisted of patients who underwent CD using monofilament sutures for uterine closure (0-Monocryl®, Ethicon) and subcuticular closure (3-0 Opepolyx®, Alfresa, Tokyo, Japan).

Results

White blood cell count on post-operative day 1 was statistically lower in the barbed suture group (p=0.01), while there were no other significant differences between the two groups.

Conclusion

Barbed sutures can be used without major complications in patients who have undergone CD, including high-risk pregnancies.

## Introduction

Cesarean delivery (CD) is one of the most common medical procedures performed on women all over the world, and it is essential for healthcare providers to keep improving operative techniques, including medical devices, in order to achieve better patient outcomes and decreased maternal morbidity [1]. The knotless barbed suture provides fast and easy closure of surgical incisions of various organs, and several studies have reported the advantages of the knotless barbed suture in CD in regard to the duration of hysterotomy closure and the overall reduction of additional sutures needed for appropriate hemostasis [2-5]. Stratafix PDS Plus (Ethicon, Somerville, NJ, USA) is one of the common barbed suture strings. Since Stratafix is coated with triclosan, it brings more favorable outcomes in surgical site infection (SSI) [6]. However, most of these reports have included relatively low-risk pregnancies, excluding cases of premature rupture of membranes, active labor, hypertensive disorders in pregnancy, and abnormal placentation [2-4]. The aim of this study was to investigate the short-term outcomes of knotless barbed sutures compared to monofilament sutures used for both closures of myometrium and subcuticular tissues in patients with various operative indications and who underwent CD.

## Materials and methods

A case-control study was conducted at the Osaka City University Graduate School of Medicine, Osaka, Japan. The patients were divided into two groups. The barbed suture group consisted of patients who underwent CD using a barbed suture for uterine closure (0 Stratafix® Spiral PDS Plus) and subcuticular closure (4-0 Stratafix® Spiral PDS Plus) between December 2019 and December 2020. The non-barbed group consisted of patients who underwent CD using a monofilament suture for uterine closure (0-Monocryl®, Ethicon, Somerville, NJ, USA) and subcuticular closure (3-0 Opepolyx®, Alfresa, Tokyo, Japan) between October 2018 and October 2019. Patients with multiple pregnancies were excluded from this study.

All patients received prophylactic intravenous antibiotics (Flomoxef sodium, Shionogi, Osaka, Japan) before the initial skin incision to reduce the rate of SSI. Povidone iodine or chlorhexidine gluconate was used for the disinfection of the abdominal surgical site and vagina. The type of anesthesia was determined by the anesthesiologist. Abdominal entries were made by a Pfannenstiel or vertical incision, and a low transverse uterine incision was then made. Vertical skin incision was selected when the previous operation scar was vertical or massive bleeding during CD was expected. Following the delivery of the fetus and placenta, the uterine incision was sutured in a double-layer technique either with a barbed suture or a non-barbed suture. When additional sutures were required for sufficient hemostasis of the uterine incision, 0 PDS Plus (Ethicon) was used in a figure-of-eight suture for both groups. Hyaluronic acid/carboxymethylcellulose (Seprafilm®, Baxter, Deerfield, IL, USA) was placed on the uterine closure site to prevent post-operative adhesion in cases with intact membranes [7]. The peritoneum was left open, and the facia was sutured continuously with monofilament (0 Monodiox®, Alfresa). For subcuticular closure, 4-0 Stratafix Spiral PDS Plus was used in the barbed suture group, and 3-0 Opepolyx was used in the control group. The use of devices for skin closure was left to each obstetrician’s discretion.

Statistical analysis was performed using SPSS software Version 24.0 (IBM Corp., Armonk, NY). Categorical data were presented as numbers and percentages, while continuous valuables were presented as mean and standard deviation. The chi-squared test was used to analyze categorical data, and the Mann-Whitney U test was used to analyze continuous valuables. P-values of <0.05 were considered significant.

This study was approved by the Ethical Committee of Osaka Metropolitan University Graduate School of Medicine (approval number: 2022-147).

## Results

A total of 221 women were included in the barbed group, and 211 were included in the non-barbed group. Table 1 shows maternal characteristics, indications of CD, and post-operative outcomes. The rate of pre-operative obstetrical complications was higher in the barbed group (52.9% vs 39.8%, p=0.003). Moreover, patients with fever ≥ 38.0°C were observed in five cases in the barbed group and four cases in the non-barbed group (Table 2). Among them, endometritis was observed in three cases in the barbed suture group and four cases in the control group. Two other cases in the barbed group with fever were diagnosed with mastitis or urinary tract infection. All patients with endometritis were treated with antibiotics, and no further invasive treatment was needed. White blood cell (WBC) count on post-operative day (POD) 1 was statistically lower in the study group (p=0.01), while WBC count on POD5 was also lower; however, the difference was not statistically different (p=0.052). There was no significant difference in CRP on POD1 and POD5 between the two groups.

**Table 1:** Maternal characteristics and indications of cesarean delivery CD, cesarean delivery; HELLP, hemolysis, elevated liver enzymes, and low platelet count

	Barbed suture (N=219)	Control (N=211)	p-Value
Maternal characteristics			
Age	34.0±5.8	34.5±5.5	0.55
Gestational age at delivery (week)	36.9±2.8	37.4±2.4	0.086
Assisted reproductive technology	48 (21.9%)	59 (28.0%)	0.14
Body mass index (kg/m^2^)	26.0±3.9	25.7±4.1	0.2
Primary CD	155 (70.1%)	138 (65.4%)	0.23
Pre-operative obstetrical complications	118 (52.9%)	84 (39.8%)	0.003
Emergent CD	117 (52.9%)	116 (55.0%)	0.89
Active labor	80 (36.2%)	84 (39.8%)	0.48
Rupture of membranes	65 (29.4%)	58 (26.2%)	0.61
Glucose intolerance	16 (7.6%)	20 (9.1%)	0.56
Indication of CD			
Previous CD	63	72	0.14
Prior myomectomy	8	7	0.53
Non-reassuring fetal status	39	37	0.52
Arrest of labor	34	29	0.35
Fetal malpresentation	20	22	0.39
Cephalopelvic disproportion	2	2	0.67
Placental abruption	4	4	0.62
Placenta previa	18	17	0.55
Vasa previa	10	3	0.050
Hypertensive disorders of pregnancy	15	11	0.31
HELLP syndrome	2	2	0.67
Eclampsia	0	1	0.49
Other	4	4	0.62

**Table 2:** Post-operative outcomes of cesarean delivery CD, cesarean delivery; CRP, C-reactive protein; POD, post-operative day; WBC, white blood cell

	Barbed suture (N=219)	Control (N=211)	p-Value
Operation time (min)	57±14	56±17	0.76
Blood loss (mL)	870±634	890±620	0.44
Fever (≥ 38.0°C) after CD	5 (2.3%)	4 (1.9%)	0.78
WBC on POD1 (x100/uL)	123.8±41.4	129.5±36.2	0.01
WBC on POD5 (x100/uL)	60.6±46.5	65.8±22.1	0.052
CRP on POD1	5.03±3.11	4.91±3.19	0.38
CRP on POD5	2.51±1.70	2.39±2.04	0.12

## Discussion

The present study is one of the largest scale studies to demonstrate the capability of using barbed sutures in CD including high-risk pregnancies without major complications. Less knots were made in the barbed suture group, possibly resulting in a favorable outcome to reduce the chance of infection at the suture site. Stratafix is coated with triclosan, which is recommended for the prevention of infection by the Centers for Disease Control and Prevention (CDC) and the American College of Surgeons Surgical Infection Society [8,9], and this manufactural process might have had a positive effect on the prevention of inflammation. Our study also showed that barbed sutures might be safely used even for high-risk pregnancies such as abnormal placentation or hypertensive disorders in pregnancy, both of which can often lead to post-partum hemorrhage (PPH) [10] and the chronic imbalance of coagulation and fibrinolysis [11]. One of the benefits of barbed sutures is a reduction in local tensile strain, which may help avoid tissue hypoxia and its resulting necrosis and infection [12,13]. These advantages will reduce the burden on the uterus in high-risk pregnancies where additional hemostatic procedures, such as uterine artery embolization and/or uterine balloon insertion to control PPH, are needed [14].

Some authors expressed concerns about the use of barbed sutures. Firstly, bowel obstruction and perforation might be caused by the proximity of their sharp barbs [15]. In the present study, however, we showed that post-operative ileus did not increase in the barbed suture group, and this observation is in line with previous studies [2-5]. Secondly, the significantly longer duration of barbed suture absorption (180 and 91-119 days for barbed suture and Monocryl, respectively) might have some possible effects, such as prolonged inflammatory reaction and/or ischemic damages, on long-term wound healing [5,16]. These issues should be meticulously investigated in the future by larger randomized controlled trials.

## Conclusions

Barbed suture can be used for wound closure in CD without severe post-operative complications. Further investigation is necessary to elucidate the advantage of Stratafix in CD operation.

## References

[1] Wise J (2018). Alarming global rise in caesarean births, figures show. BMJ.

[2] Peleg D, Ahmad RS, Warsof SL, Marcus-Braun N, Sciaky-Tamir Y, Ben Shachar I (2018). A randomized clinical trial of knotless barbed suture vs conventional suture for closure of the uterine incision at cesarean delivery. Am J Obstet Gynecol.

[3] Zayed MA, Fouda UM, Elsetohy KA, Zayed SM, Hashem AT, Youssef MA (2019). Barbed sutures versus conventional sutures for uterine closure at cesarean section; a randomized controlled trial. J Matern Fetal Neonatal Med.

[4] Grin L, Namazov A, Ivshin A (2019). Barbed versus conventional suture for uterine repair during caesarean section: a randomized controlled study. J Obstet Gynaecol Can.

[5] Meyer R, Sharon N, Sivan E (2019). Maternal morbidity following caesarean deliveries with barbed suture for uterine closure. Arch Gynecol Obstet.

[6] Ruiz-Tovar J, Llavero C, Jimenez-Fuertes M, Duran M, Perez-Lopez M, Garcia-Marin A (2020). Incisional surgical site infection after abdominal fascial closure with triclosan-coated barbed suture vs triclosan-coated polydioxanone loop suture vs polydioxanone loop suture in emergent abdominal surgery: a randomized clinical trial. J Am Coll Surg.

[7] Zeng Q, Yu Z, You J, Zhang Q (2007). Efficacy and safety of Seprafilm for preventing postoperative abdominal adhesion: systematic review and meta-analysis. World J Surg.

[8] Ban KA, Minei JP, Laronga C (2017). American College of Surgeons and Surgical Infection Society: Surgical Site Infection Guidelines, 2016 Update. J Am Coll Surg.

[9] Berríos-Torres SI, Umscheid CA, Bratzler DW (2017). Centers for Disease Control and Prevention Guideline for the Prevention of Surgical Site Infection, 2017. JAMA Surg.

[10] Oyelese Y, Smulian JC (2006). Placenta previa, placenta accreta, and vasa previa. Obstet Gynecol.

[11] Dusse LM, Rios DR, Pinheiro MB, Cooper AJ, Lwaleed BA (2011). Pre-eclampsia: relationship between coagulation, fibrinolysis and inflammation. Clin Chim Acta.

[12] Jonsson T, Högström H (1992). Effect of suture technique on early healing of intestinal anastomoses in rats. Eur J Surg.

[13] Högström H, Haglund U, Zederfeldt B (1990). Tension leads to increased neutrophil accumulation and decreased laparotomy wound strength. Surgery.

[14] American College of Obstetricians and Gynecologists (2006). ACOG Practice Bulletin: Clinical Management Guidelines for Obstetrician-Gynecologists Number 76, October 2006: postpartum hemorrhage. Obstet Gynecol.

[15] Clapp B, Klingsporn W, Lodeiro C, Wicker E, Christensen L, Jones R, Tyroch A (2020). Small bowel obstructions following the use of barbed suture: a review of the literature and analysis of the MAUDE database. Surg Endosc.

[16] Greenberg JA (2010). The use of barbed sutures in obstetrics and gynecology. Rev Obstet Gynecol.

